# Wavefront aberrations and retinal image quality in different lenticular opacity types and densities

**DOI:** 10.1038/s41598-017-15245-4

**Published:** 2017-11-10

**Authors:** Cheng-Zhe Wu, Hua Jin, Zhen-Nv Shen, Ying-Jun Li, Xun Cui

**Affiliations:** 1 Department of Ophthalmology, Affiliated Hospital of Yanbian University, Yanji Jilin, 133-000 China; 2 Department of Physiology and Pathophysiology, School of Medical Sciences, Yanbian University, Yanji Jlin, 133-002 China

## Abstract

To investigate wavefront aberrations in the entire eye and in the internal optics (lens) and retinal image qualities according to different lenticular opacity types and densities. Forty-one eyes with nuclear cataract, 33 eyes with cortical cataract, and 29 eyes with posterior subcapsular cataract were examined. In each group, wavefront aberrations in the entire eye and in the internal optics and retinal image quality were measured using a raytracing aberrometer. Eyes with cortical cataracts showed significantly higher coma-like aberrations compared to the other two groups in both entire eye and internal optic aberrations (*P* = 0.012 and *P* = 0.007, respectively). Eyes with nuclear cataract had lower spherical-like aberrations than the other two groups in both entire eye and internal optics aberrations (*P* < 0.001 and *P* < 0.001, respectively). In the nuclear cataract group, nuclear lens density was negatively correlated with internal spherical aberrations (*r* = −0.527, *P* = 0.005). Wavefront technology is useful for objective and quantitative analysis of retinal image quality deterioration in eyes with different early lenticular opacity types and densities. Understanding the wavefront optical properties of different crystalline lens opacities may help ophthalmic surgeons determine the optimal time to perform cataract surgery.

## Introduction

The accurate assessment of visual function interference by lens opacities can help ophthalmic surgeons choose the best time to perform cataract surgery. In the early stages of cataract formation, patients may suffer from decreased visual quality (light scattering, polyopia, etc.), whereas visual acuity in standard conditions is still preserved^1^.The deterioration of visual function in these patients cannot be entirely explained by spherical or cylindrical refractive errors^2^. Contrast sensitivity declines with age, even in the absence of ocular pathologies, such as manifest cataract, glaucoma, or macular degeneration^3^. This decline in visual capability likely involves deterioration of retinal image qualities due to changes in higher-order aberrations of the crystalline lens^4–6^. It is also reported that a loss of contrast sensitivity closely relates to the density of nuclear cataract, as measured by Scheimpflug images^7^.

The isolation of aberrations that result from a crystalline lens is essential to determining the effects of crystalline lens opacities on wavefront aberration; however, baseline wavefront data on isolated internal optics (lens) of the eye have not been fully investigated, because most aberrometers that used previous studies only measures the total ocular wavefront aberrations. The iTrace (Tracey Technologies), which is a commercially available visual function analyzer that combines ray-tracing aberrometry and corneal topography, is able to sequentially projects 256 near-infrared laser beams into the eye in a specific scanning pattern^8^. The aberrations of the internal optics are directly measured by locating the spots on the retina within milliseconds^9^. In addition to optical aberration data, the iTrace provides the modulation transfer function (MTF) curves from 0–30 cycles/degree (cpd). The MTF obtained from double-pass line spread function (LSF) is another parameter to measure the retinal image qualities objectively because LSF flattens as nuclear opalescence increases^10^. Alió *et al*.^11^ found a linear evolution in the optical quality (intraocular aberrations and ocular MTF) between eyes with clear lenses older than 40 years and eyes with a low grade of cataract.

In this study, we investigated the entire eye and internal optical wavefront aberrations and retinal image qualities that are induced by different lenticular opacity types and densities using the iTrace visual function analyzer. We also investigated the correlation between nuclear lens density measured by scheimpflug images and the internal optical aberration in nuclear cataracts.

## Materials and Methods

The study was approved by the Affiliated Hospital, Yanbian University Medical College Institutional Review Board. One hundred and three patients (103 eyes) with age-related cataract were recruited from January to June 2015 at Affiliated Hospital, Yanbian University. Patients with a history of ocular disease (pterygium, corneal opacities, corneal irregularities, glaucoma, retinal pathology, or high myopia) and a history of intraocular surgery or laser treatment were excluded. The Institutional Review Board approval was obtained and the study was conducted in accordance with the declaration of Helsinki. Written informed consent was obtained from all of the participants.

All patients had a complete ophthalmic examination including a measurement of visual acuity, non-contact tonometry, slit lamp biomicroscopy, and dilated fundus examination. The subjects had a spherical equivalent (SE) between +3.00 and −3.00 diopters (D), and best corrected visual acuity (BCVA) better than 0.3 logMAR units. The degree of cataract was classified according to the Lens Opacities Classification System III (LOCS III)^12^ system using a slit lamp biomicroscope after pupil dilatation with 0.5% or 1.0% tropicamide. Forty-two eyes (36%) had nuclear cataracts (NO 2–4, C 0–1, and P 0–1 on the LOCS III scale), and 39 eyes (34%) had cortical opacifications (C 2–4, NO 0–1 and P 0–1). Thirty-five eyes (30%) had posterior subcapsular cataracts (P 2–4, C 0–1, and NO 0–1).

Additional assessments were performed using an iTrace aberrometer (Tracey Technologies, Houston, TX, USA) and Scheimpflug images (Oculus Pentacam, Dutenhofen, Germany) by a single examiner (Y-J.L.). For each eye, measurements were repeated at least three times to obtain a well-focused, properly aligned image of the eyes. Before measurements, the pupil was dilated (>6.0 mm) with 0.5% or 1.0% tropicamide.

Wavefront analysis was conducted using the iTrace aberrometer in a dark room. The entire eye and corneal higher-order aberrations were measured with a 6.0 mm pupil. The wavefront aberrations in the cornea were calculated based on topographical data, and the aberrations in the internal optics of the eye were calculated by subtracting the aberrations in the cornea from those in the entire eye measured by the ray-tracing aberrometer using the built-in program. The higher-order aberrations was analyzed by expanding the results that were obtained using Zernike polynomials and the root mean square (RMS) values of the total higher-order (3rd to 6th order), third-order and fourth-order aberrations, spherical-like (Z4.0 and Z6.0) aberrations, and coma-like (Z3.-1, Z3.1, Z5.-1, and Z5.1) aberrations. In addition, we analyzed MTF curves for each patient from only higher-order aberrations to eliminate the effects of lower-order aberrations for the 6.0 mm pupil (e.g., defocus and astigmatism).

**Figure 1:**
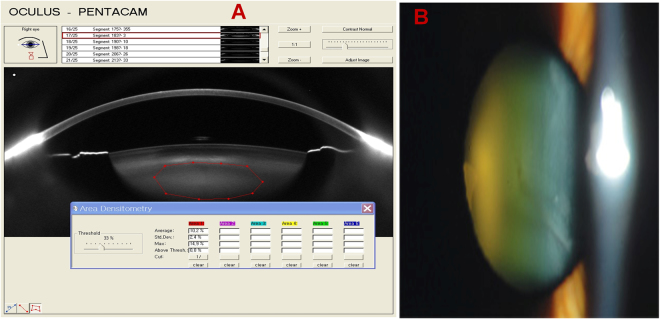
Pentacam Scheimpflug image of a nuclear cataract. (**A**) Average nuclear lens density in the region of interest, marked by the elliptical mask and (**B**) digital slit-lamp photograph of the same lens.

**Figure 2:**
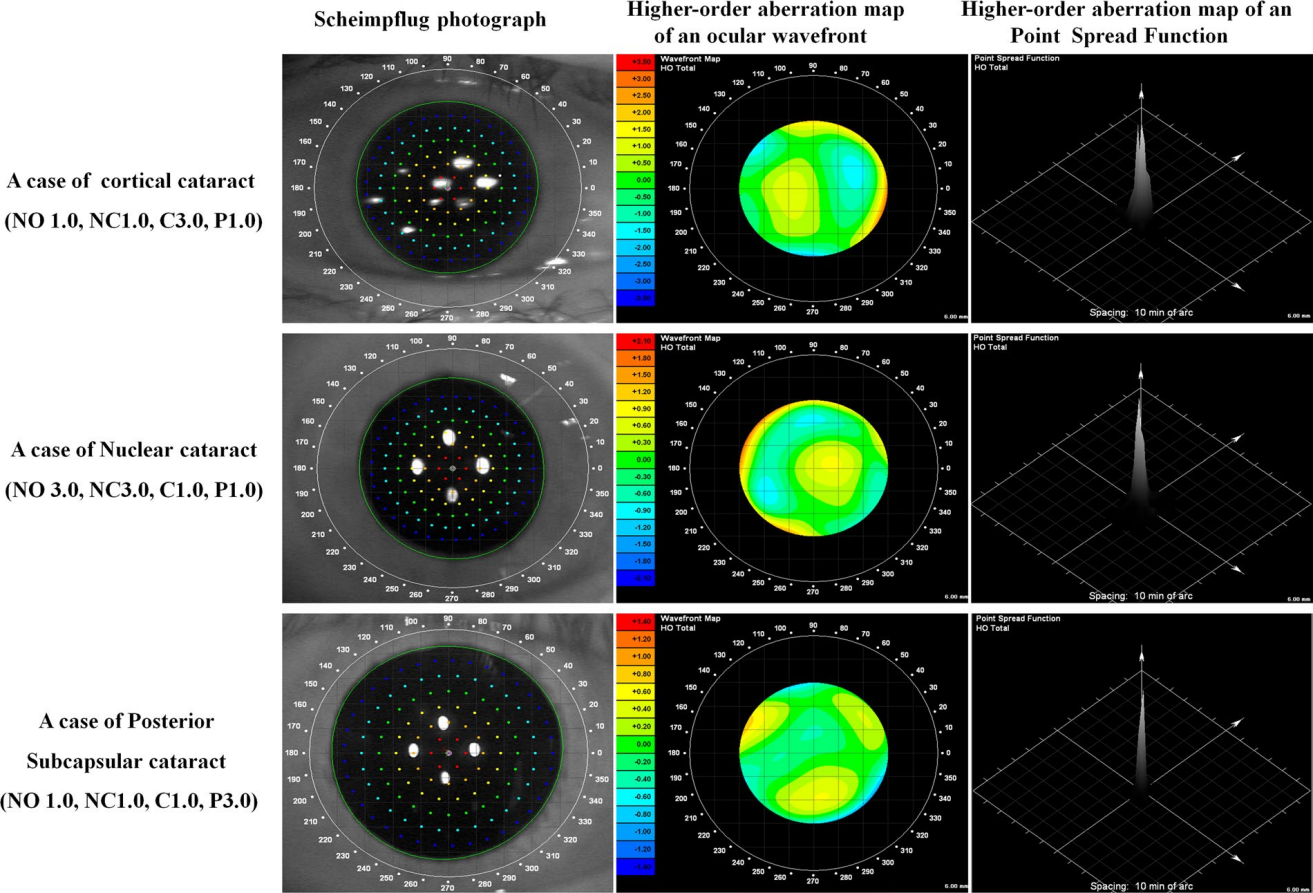
The higher-orderaberration map of ocular Modulation Transfer Function(MTF) by cataract group.

Nuclear lens density was measured using Pentacam Scheimpflug images. It has been established that certain lens regions are more representative of the opacification and change in severity of nuclear sclerosis (or lens density) than others^13^. Using individual images, the program quantified lens density on a scale of 0 to 100 (0 = no cloudiness; 100 = completely opaque lens) (Fig. 1). A Scheimpflug image of the lens was exported to ImageJ software for measuring the nuclear lens density^14–16^. In the nuclear cataract group, the relationship between nuclear lens density and intraocular aberrations and also the relationship between nuclear lens density and MTF were examined. Figure 2 shows the higher-orderaberration map of ocular Modulation Transfer Function(MTF) by cataract group.

Statistical analysis was performed using SPSS version 12 (SPSS, Inc. Chicago, IL, USA). The comparisons of wavefront variables among 3 groups were performed using the analysis of variance test (ANOVA), and the differences therein were calculated using Duncan’s multiple-range test. The relationship between nuclear lens density and internal optics aberration was analyzed using the Spearman rank correlation coefficient. A *P* value less than 0.05 was considered statistically significant.

## Results

In this study, 103 eyes of 103 patients (53 men, 50 women) were evaluated. The mean age of the patients was 65.8 ± 6.3 years (range 52 to 76 years). Table 1 shows the demographic data of the patients by groups. There were no statistically significant differences between groups in age, best spectacle-corrected visual acuity (BSCVA) and corneal asphericity (Q-values) (*P* = 0.856, *P* = 0.579 and *P* = 0.712, respectively).

**Table 1:** Patient characteristics for the three groups.

	Mean ± SD			*P-value*
	Cortical	Nuclear	PSC	
Number (M/F)	33 (16/17)	41 (22/19)	29 (15/14)	0.891
Age (years)	65.03 ± 5.26	66.52 ± 6.41	65.82 ± 6.65	0.756
BSCVA (log MAR)	0.25 ± 0.07	0.24 ± 0.09	0.21 ± 0.05	0.579
Corneal astigmatism (D)	0.96 ± 0.68	1.17 ± 0.90	1.04 ± 0.73	0.450
Q-value	−0.16 ± 0.09	−0.15 ± 0.10	−0.15 ± 0.08	0.712
Defocus	2.15 ± 1.53	1.86 ± 1.13	1.91 ± 1.26	0.341
Astigmatism	1.05 ± 0.54	0.89 ± 0.63	0.97 ± 0.41	0.687

PSC posterior subcapsular cataract, BSCVA best spectacle-corrected visual acuity, Q-value corneal asphericity.

Table 2 shows the wavefront aberrations in the entire eye, internal optics of the eye, and cornea with a 6.0 mm pupil in the cortical, nuclear, and posterior subcapsular cataract groups. There was no difference in corneal aberrations among the groups. In the entire eye and internal optics of the eye, the cortical and nuclear cataract groups had statistically significantly lower total HOA than the posterior subcapsular group (*P* = 0.028 and *P* = 0.009, respectively). The cortical cataract group had statistically significantly higher coma-like aberrations than the nuclear and posterior subcapsular groups (*P* = 0.012 and *P* = 0.007, respectively). The nuclear cataract group had statistically significantly lower spherical aberrations (negative shift) than the other two groups (*P* < 0.001 and *P* < 0.001, respectively).

**Table 2:** Entire eye, internal optics, and corneal aberrations by cataract group for a 6.0 mm pupil diameter.

Category	Mean ± SD			*P- value*
	Cortical	Nuclear	PSC	
Entire Eye (μm)				
HOA RMS	0.76 ± 0.37	0.55 ± 0.31	0.89 ± 0.32	0.028*
Coma	0.43 ± 0.21	0.26 ± 0.17	0.31 ± 0.12	0.012*
Spherical aberration	0.24 ± 0.15	0.11 ± 0.32	0.29 ± 0.11	<0.001*
Trefoil	0.27 ± 0.23	0.29 ± 0.21	0.32 ± 0.23	0.162
Internal Optics (μm)				
HOA RMS	0.95 ± 0.46	0.71 ± 0.54	1.13 ± 0.43	0.009*
Coma	0.76 ± 0.42	0.51 ± 0.23	0.61 ± 0.24	0.007*
Spherical aberration	0.08 ± 0.14	−0.35 ± 0.41	0.11 ± 0.17	<0.001*
Trefoil	0.45 ± 0.31	0.49 ± 0.23	0.57 ± 0.36	0.095
Cornea (μm)				
HOA RMS	0.49 ± 0.11	0.51 ± 0.13	0.47 ± 0.15	0.461
Coma	0.21 ± 0.10	0.22 ± 0.13	0.19 ± 0.11	0.713
Spherical aberration	0.28 ± 0.06	0.30 ± 0.05	0.27 ± 0.08	0.598
Trefoil	0.21 ± 0.12	0.20 ± 0.09	0.19 ± 0.07	0.422

PSC posterior subcapsular cataract, HOA higher-order aberration, RMS root mean square, *P < 0.05.

**Figure 3:**
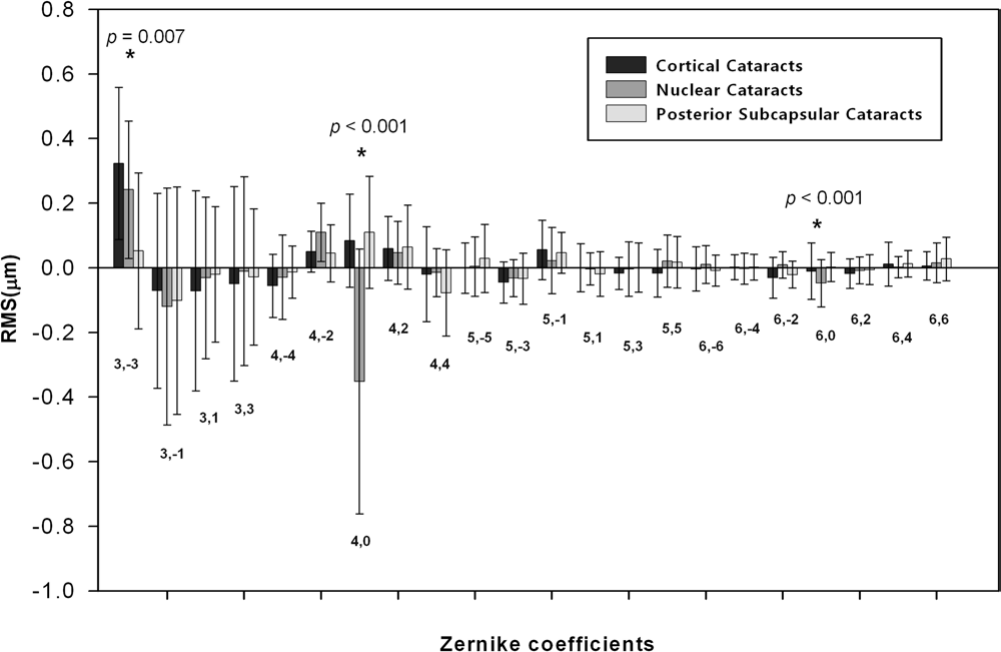
Mean Zernike coefficients of the internal optics (pupil size: 6 mm, third to sixth order coefficients) in the cortical, nuclear, and posterior subcapsular cataract groups. *P < 0.05, error bars represent the SD.

Figure 3 shows higher-order Zernike coefficients of the internal optics (3rd to 6th order) in the three groups. The nuclear cataract group had statistically significantly lower internal spherical aberrations (Zernike coefficient, Z4.0 and Z6.0) than the cortical and posterior subcapsular cataract groups (*P* < 0.001 and *P* < 0.001, respectively). The cortical cataract groups had statistically significantly higher trefoil (Z3.-3) than the other two groups (*P* = 0.007). Other HOAs showed no significant differences.

**Figure 4:**
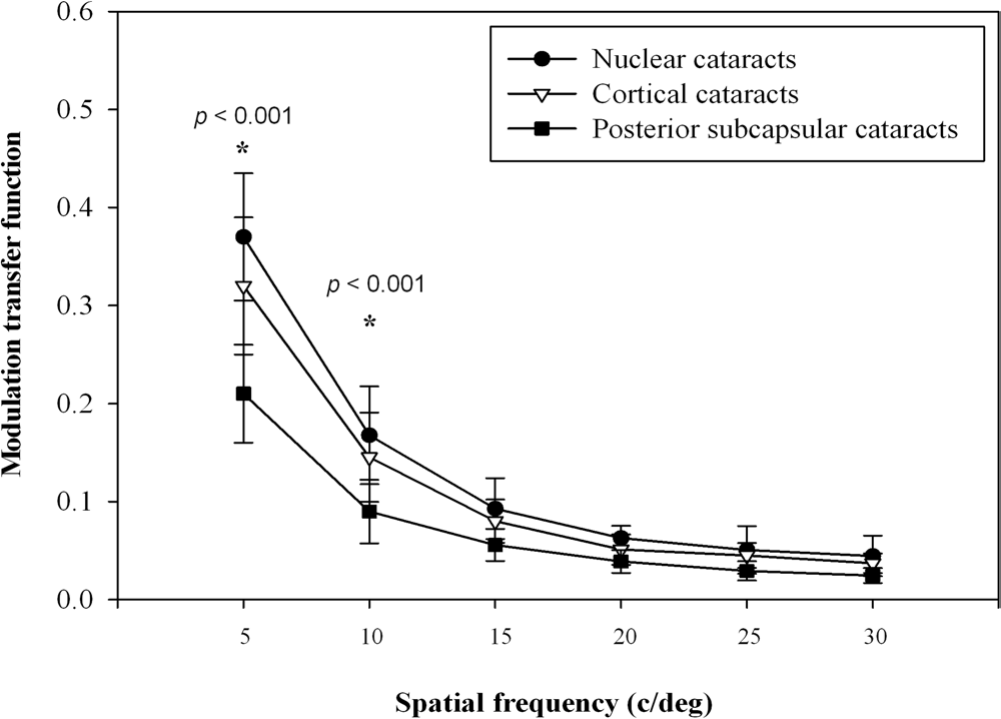
Modulation transfer function curves according to cataract group for a 6 mm pupil diameter at different spatial frequencies. *P < 0.05, error bars represent the SD.

Figure 4 shows the MTF curves under the 6 mm pupil in cortical, nuclear, and posterior subcapsular cataract groups. The posterior subcapsular cataract group had lower performance than cortical and nuclear cataract groups at 5 and 10 cpd (*P* < 0.001 and *P* < 0.001, respectively). No statistically significant difference was found between the cortical and nuclear cataract groups at all spatial frequencies.

**Figure 5:**
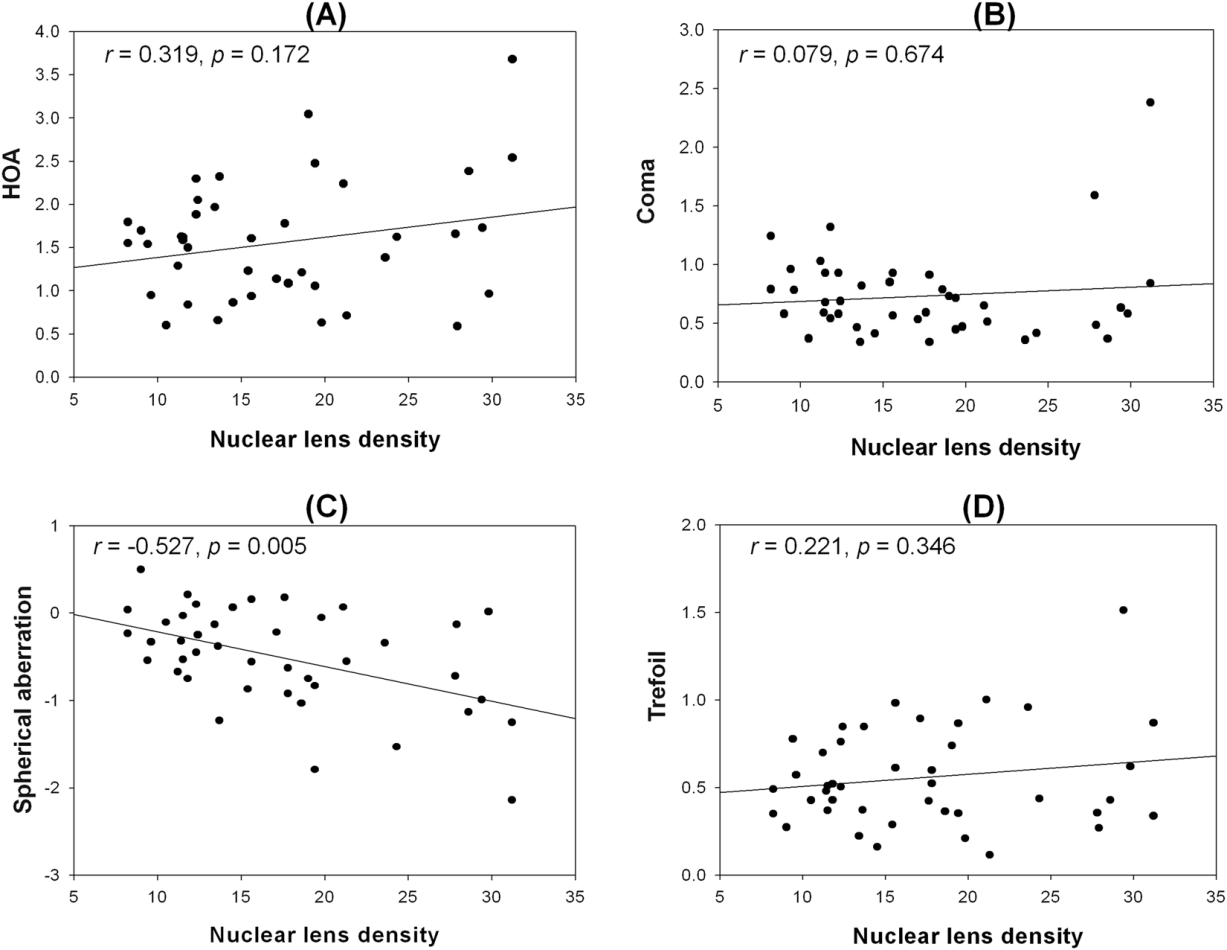
Relationship between nuclear lens density and internal optics aberrations in the nuclear cataract group. (**A**) RMS HOA, (**B**) coma, (**C**) spherical aberration, (**D**) trefoil. The correlation is significant; *r* and *P* values were calculated by Spearman rank correlation.

Figure 5 shows the relationships between nuclear lens density and internal optics aberrations in the nuclear cataract group. The RMS of HOAs, coma, and trefoil had no significant correlation with the nuclear lens density (*r* = 0.319, *P* = 0.172; *r* = 0.079, *P* = 0.674; and *r* = 0.221, *P* = 0.346, respectively). However, as the nuclear lens density increased, the spherical aberration in the internal optics of the eye showed a negative shift (*r* = −0.527, *P* = 0.005).

**Figure 6:**
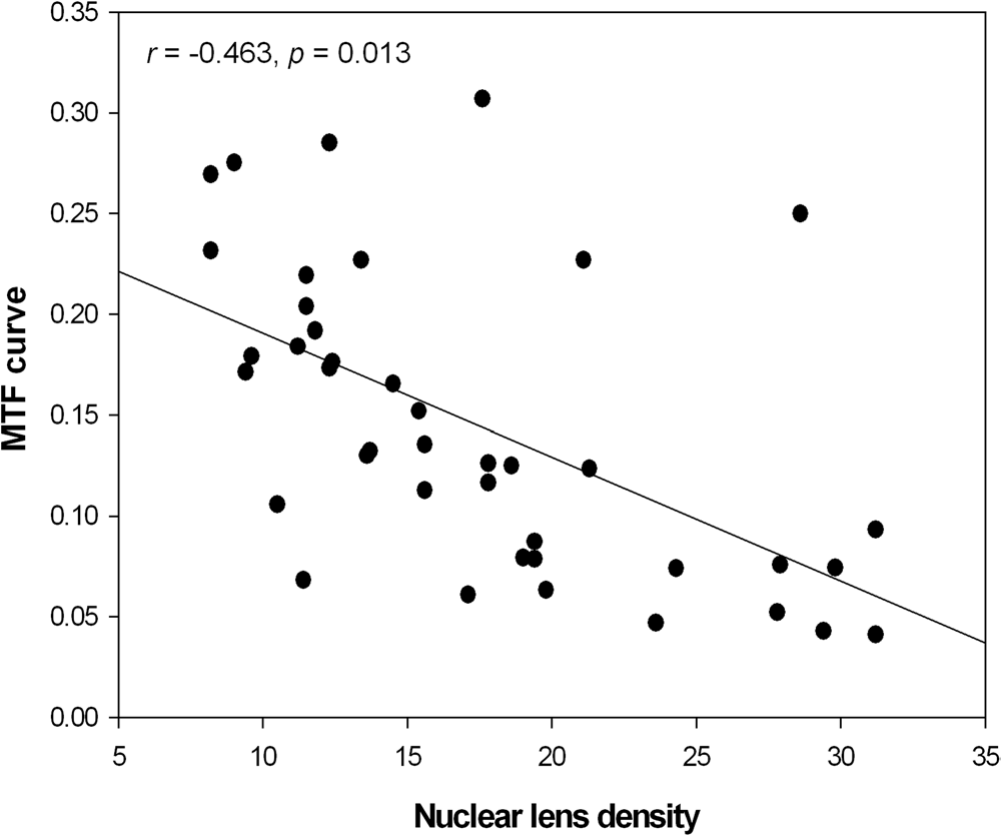
Relationship between average height from the modulation transfer function curve (modulation between 0 and 30 cpd) and nuclear lens density in the nuclear cataract group. A negative linear correlation was found (*r* = −0.463, *P* = 0.013).

In Fig. 6, nuclear lens density was negatively correlated with the average height for each MTF curve (modulation between 0 and 30 cpd) in the nuclear cataract group (*r* = −0.463, *P* = 0.013).

## Discussion

Wavefront technology is a unique tool for measuring and quantifying the aberrations produced by the eye as a whole optical system^17^. Amano *et al*.^18^ found that ocular spherical aberration and coma increase with age. The increase of spherical aberration is related to internal optical changes, presumably lenticular opacification, whereas the increase in coma is likely due to age-related changes in the cornea. Recently, the iTrace aberrometer was developed in order to measure optical aberration in the entire eye and in the internal optics^9,19,20^.

In this study we analyzed entire eye and internal optics aberrations in different types of senile cataracts using the iTrace aberrometer. Visual image quality was also assessed using the MTF curve that was provided by the iTrace.

Applegate *et al*.^21,22^ reported that aberrations near the center of the Zernike table (e.g., coma, trefoil, and spherical aberrations) tend to more significantly affect visual quality than those at the periphery of the table. Sachdev *et al*.^23^ found that cortical cataract predominantly increases coma, whereas nuclear opacification increases spherical aberration. Rocha *et al*.^24^ also described that coma predominates in the cortical cataract group, and spherical aberration predominates in the nuclear cataract group. In agreement with their studies, the cortical cataract group had significantly higher coma-like aberrations and the nuclear cataract group had significantly higher spherical-like aberrations in the internal optics of the eye, whereas corneal aberrations showed no statistical differences among the three groups. However, the polarity of the intraocular spherical aberrations that were investigated in this study are opposite to that of former studies; the nuclear cataract group exhibited negative spherical aberration.

On the other hand, Lee *et al*.^2^ found that spherical aberrations in internal optics negatively correlate with grade of nuclear cataract, which is consistent with our findings. Kuroda *et al*.^25^ described that in nuclear cataracts, the polarity of spherical aberrations is negative because the wavefront is delayed when the ray travels inside the higher refractive index hard nucleus, whereas normal lenses have positive spherical aberrations.

Stifter *et al*.^26^ compared contrast acuity scores in early, intermediate, and advanced nuclear, cortical, and posterior subcapsular cataract groups. In their study, posterior subcapsular cataract showed significantly reduced contrast acuity scores compared to other groups. The comparably strong influence of posterior subcapsular cataract on functional vision may result from the typical morphologies of posterior subcapsular lens opacities, which obscures the eye’s nodal point and may result in central visual loss and an increase in HOA^27^. These findings are consistent with our study. In this study, the posterior capsular cataract group had the lowest MTF curve and also statistically significantly higher HOAs compared to the cortical and nuclear cataract groups. The decreased functional vision in posterior subcapsular cataracts may be explained by higher wavefront aberrations and a lower retinal image quality by MTF.

These findings suggest that wavefront analysis provides clinically relevant information about functional visual performance and can be included in the clinical evaluation of cataract patients so as to determine the best time to perform surgery. In this regard, surgery on posterior capsular cataracts should be considered earlier than other types of cataracts.

Nuclear cataract can decrease visual function by affecting contrast sensitivity. Deterioration in contrast sensitivity can be explained in terms of scatter and an increased in HOAs^28^. In the current study, we also evaluated the relationship of nuclear lens density to wavefront optical properties in nuclear cataract patients.

We conducted quantitative lens optical density measurements in nuclear cataract patients using the Pentacam Scheimpflug system, which is correlated with LOCS III grading^29^. The correlation between nuclear lens density and the RMS of HOA, coma, and trefoil in the internal optics of the eye was not significant in the nuclear cataract group. However, a negative and significant linear correlation was found between intraocular spherical aberration Z(4.0) and nuclear lens density. In addition, nuclear lens density negatively correlated with the intraocular spherical aberration in the nuclear cataract group. To our knowledge, this is the first study that the objective lens density obtained using standardized method of the Pentacam Schimpflug system to investigate the relationship between nuclear lens density and wavefront aberrations.

In addition, the MTF that accompanied the aforementioned commercially available ray-tracing aberrometer has not been previously used to measure visual quality according to lens opacities. This finding supports the clinical relevance of reporting decreased visual quality in patients with early nuclear cataracts, although their visual acuities may still be preserved. Scheimpflug photography and MTF evaluation may provide useful tools in the quantitative evaluation of early stage of cataract which could not be detected by standard visual acuity tests.

In conclusion, wavefront technology is useful in the measurement of the deterioration of image quality objectively and quantitatively and is supposed to provide clinically relevant information about the functional visual performance in different lenticular opacity types and densities. Therefore, we recommend assess the waverfront aberration in periodic follow-up of mild cataract, which may help ophthalmic surgeons to determine the optimal time to perform the surgery.
